# An NF-κB and Slug Regulatory Loop Active in Early Vertebrate Mesoderm

**DOI:** 10.1371/journal.pone.0000106

**Published:** 2006-12-27

**Authors:** Chi Zhang, Timothy F. Carl, Evan D. Trudeau, Thomas Simmet, Michael W. Klymkowsky

**Affiliations:** 1 Molecular, Cellular, and Developmental Biology, University of Colorado, Boulder, Colorado, United States of America; 2 Institute of Pharmacology of Natural Products and Clinical Pharmacology, University of Ulm, Ulm, Germany; Max Planck Institute of Molecular Cell Biology and Genetics, Germany

## Abstract

**Background:**

In both *Drosophila* and the mouse, the zinc finger transcription factor Snail is required for mesoderm formation; its vertebrate paralog Slug (Snai2) appears to be required for neural crest formation in the chick and the clawed frog *Xenopus laevis*. Both Slug and Snail act to induce epithelial to mesenchymal transition (EMT) and to suppress apoptosis.

**Methodology & Principle Findings:**

Morpholino-based loss of function studies indicate that Slug is required for the normal expression of both mesodermal and neural crest markers in *X. laevis*. Both phenotypes are rescued by injection of RNA encoding the anti-apoptotic protein Bcl-xL; Bcl-xL's effects are dependent upon IκB kinase-mediated activation of the bipartite transcription factor NF-κB. NF-κB, in turn, directly up-regulates levels of *Slug* and *Snail* RNAs. Slug indirectly up-regulates levels of RNAs encoding the NF-κB subunit proteins RelA, Rel2, and Rel3, and directly down-regulates levels of the pro-apopotic Caspase-9 RNA.

**Conclusions/Significance:**

These studies reveal a Slug/Snail–NF-κB regulatory circuit, analogous to that present in the early *Drosophila* embryo, active during mesodermal formation in Xenopus. This is a regulatory interaction of significance both in development and in the course of inflammatory and metastatic disease.

## Introduction

The process of transforming relatively immotile epithelial cells into actively migrating mesenchymal cells, known as epithelial to mesenchymal transition (EMT), is central to a wide range of biological processes from mesoderm, mesenchyme, and neural crest formation to pathogenic fibrosis and metastasis [1]–[4]. Important players in the regulation of EMT are the zinc finger transcription factors Snail (Snai1) and its vertebrate paralog Slug (Snai2). In addition to Snail and Slug, a number of other members of the Snail family have been identified. In *Drosophila* *melanogaster* there are the Snail-like genes *Escargot* and *Worniu* [5]–[7], and the more divergent *Scratch* genes [8]. The duplication event that gave rise to *Snail*-like and *Scratch*-like genes appears to have occurred before the divergence of proteostomes and deuterostomes [9], [10].

The involvement of Snail-like proteins in EMT was first suggested by genetic studies in *Drosophila*. Mutations in *Snail* lead to the disruption of mesoderm and embryonic lethality [11]–[13]. As in *Drosophila*, mice homozygous for a null mutation in the orthologous *Snail* gene fail to form normal mesoderm and exhibit early embryonic lethality [14]. No mesodermal phenotype was observed in mice homozygous for a null mutation in *Slug* [15], [16]. The absence of Slug does lead to defects in melanocyte, hematopoietic stem cell and germ cell development, and epidermal healing [17]–[19]. Slug is expressed in the mesoderm in the chick and exposure of the early embryo to anti-sense oligonucleotides leads to defects in mesoderm emergence [20]. In *X. laevis* Slug mRNA is expressed zygotically in the dorsal mesendoderm; interference with its function, through the injection of RNAs encoding dominant negative proteins, leads to defects in the expression of organizer (*Chordin*, *Cerberus*) and ventrally (*Xwnt8*, *Xvent1*) expressed genes [21]. An important concern about such studies involves the specificity of “anti-morphic” reagents, given the known regulatory cross-talk between E-box binding Snail, Slug and basic helix-loop-helix (bHLH) transcription factors (see below).

In both *X. laevis* and the chick, Slug appears to have an essential role in neural crest formation [20], [22]–[24]. In contrast, mutation of *Slug* has no apparent effect on neural crest formation in the mouse [15]. This apparent discrepancy was initially ascribed to a swapping of *Slug* and *Snail* expression domains in the mouse [25], [26]. More recent studies, using a combination of constitutive and conditional knock out mutations, indicate that neither Slug nor Snail are required for neural crest formation in the mouse, at least in the cranial region [16].

Snail-like proteins are generally thought to act as transcriptional repressors, although Sakai et al [27] report that Slug positively regulates is own expression. Snail, Slug, and Scratch all bind to E-box sequences (CANNTG) and can antagonize the activity of bHLH proteins [8], [28]–[31]. In their role as regulators of EMT, Slug and Snail have been found to suppress expression E-cadherin and tight junction components and the forced expression of Slug disrupts adherens junctions, tight junctions, and desmosomes [32]–[39]. Slug and Snail also act as inhibitors of apoptosis [40]–[44]. Slug has been found to negatively regulate the expression of the pro-apoptotic *p53* [43] and *Puma* [45] genes. Subsequent studies have found that Slug is required for the metastasis of human melanoma cells [46] and has been implicated in lung adenocarcinoma and breast carcinoma invasiveness [47]–[49].

Sequence analysis indicate that *Slug*s are more conserved than vertebrate *Snail*s [50]. Lespinet et al [10] grouped chick (*Gallus gallus*) and *X. laevis* Snails with Slugs rather than with other vertebrate Snails. Slug and Snail have been found to be functionally similar, but not identical. For example, injection of RNA encoding Snail rescues the effects of anti-sense Slug RNA injection in *X. laevi*s [22] and “Slug and Snail can be can be functionally equivalent when tested in overexpression studies” [23]. Over-expression of Snail leads to expansion of the neural crest domain in the chick, much as observed following over-expression of Slug [51], [52]. On the other hand, the need for Snail expression in the early Drosophila embryo cannot be replaced by either Escargot or Worniu [53]. Snail and Slug differ in their ability to induce neural crest markers in *X. laevis* ectodermal explants [23], even though Slug alone has been found to rescue the effects on neural crest following the blocking of both Slug and Snail activity [see 24]. Slug appears to bind less strongly to regulatory regions in the E-cadherin protein than does Snail [38], while Slug, but not Snail, has been found to mediate genotoxin resistance in human mesothelioma cells [54]. A microarray-based analysis of MDCK epithelial cells found both common and distinct sets of genes regulated by Slug and Snail [55]. Given that Snail [56]–[59] and Slug [60] can be post-translationally regulated in terms of both stability and intracellular localization, it remains unclear whether the differences between the two proteins are intrinsic or are due to protein-specific post-translational effects.

Previous studies of Slug's role in *X. laevis* have used either anti-sense RNA [22] or dominant-negative proteins [21], [23], [24], [61], [62] to disrupt Slug expression and/or activity. As part of a study to separate the role of Slug in EMT from its role as a regulator of apoptosis, we designed a modified anti-sense DNA oligonucleotide (a morpholino) that blocks Slug expression. In the course of analyzing the ability of the anti-apoptotic protein Bcl-xL to rescue the phenotypic effects of this morpholino, we uncovered an essential role for NF-κB as a regulator of *Slug* expression in the early embryo, a regulatory interaction analogous to that observed in the early *Drosophila* embryo, and not apparently described previously in a vertebrate.

## Results

**Figure 1:**
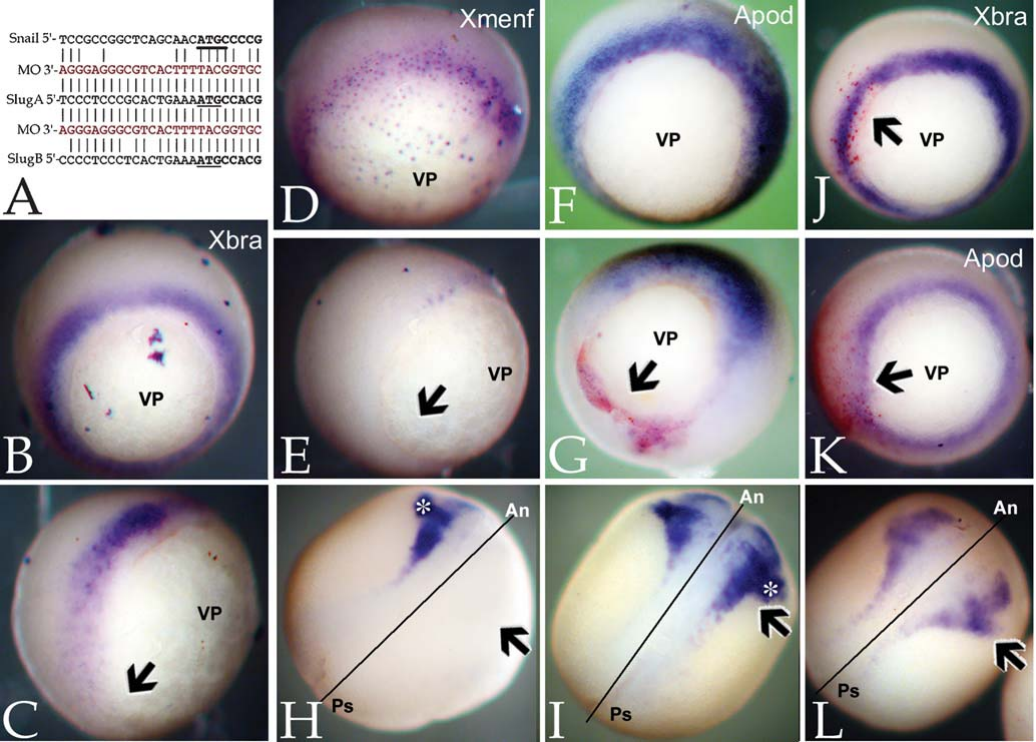
Slug morpholino effects: Panel A is a comparison of the Slug morpholino sequence (“MO”) with *X. laevis* SlugA, SlugB, and Snail RNA sequences; start codons are underlined. B–G: Injection of the Slug MO (10 ng/embryo) blocks the expression of *Xbra* (B-uninjected, C-Slug MO injected), *Xmenf* (D-uninjected, E-injected), and *Antipodean* (Apod)(F-uninjected, G-injected). Arrows (C,E,G) point to region of suppression; vegetal pole (“VP”) is indicated. In G, the red staining is due to a β-galactosidase lineage marker. H: Injection of the Slug MO into one cell of a two cell embryo blocks the expression of *Sox9* on the injected side (arrow); “*” marks otic placode domain of Sox9 expression. I: *Sox9* expression in Slug MO injected embryos is rescued by co-injection of mycGFP-Slug RNA (650 pg/embryo). In analogous studies, the effects of the Slug MO (10 ng/embryo) on Xbra (J), Apod (K) and Sox9 (L) expression were rescued by injection of Snail RNA (500 pg/embryo). In H, I and L, the line marks midline of the embryo, with anterior (“An”) and posterior (“Ps”) indicated.

Previous studies on the role of Slug in Xenopus have relied on injection of either anti-sense RNA directed against 3′ untranslated region of the SlugA mRNA [22] or RNAs encoding various dominant-negative proteins [21], [23], [24], [61], [62]. To complement these studies, we developed a morpholino (Slug MO) directed against the Slug mRNAs. There are two Slug pseudoalleles in *X. laevis*, *SlugA* and *SlugB* [63]. The Slug MO is a perfect match to the SlugA mRNA, has three mismatches to the SlugB mRNA and 12 (out of 25) mismatches with the analogous region of the Snail mRNA (FIG. 1A). The Slug MO blocked the *in vitro* translation of SlugA RNA that contained its target sequence but had no effect on the translation of mycGFP-Slug RNA, which lacks SlugA's 5′ untranslated region (data not shown).

When injected into one cell of two-cell embryos, the Slug MO (10 ng/embryo) inhibited expression of the mesodermal markers *Xbra* (FIG. 1B,C), *Xmenf* (FIG. 1D,E), and *Antipodean* (Apod)(FIG. 1F,G) in late blastula/early gastrula stage embryos. In neurula stage embryos, the Slug MO inhibited expression of *Sox9* (FIG. 1H), a marker of cranial neural crest and otic placodes [64], [65]. In later stage embryos, the Slug MO led to the loss of craniofacial cartilages and the otic vesicle (data not shown), very much as observed in embryos injected with Slug anti-sense RNA [22]. Injection of a control MO had no apparent effect on any of the markers examined (Table 1). As a control for the specificity of the Slug MO, embryos injected with Slug MO were injected with RNA encoding mycGFP-tagged Slug; both normal *Sox9* expression (FIG. 1I) and craniofacial morphology (data not shown) were rescued; injection of RNA encoding myc-GFP did not rescue either phenotype (Table 1 and data not shown). Previously, we found that injection of Snail RNA rescued the phenotypic effects of anti-sense Slug RNA injection [22]. This is also the case with the Slug morpholino; injection of 500 pg/embryo Snail RNA rescued expression of both mesodermal and neural crest markers in Slug MO injected embryos (FIG. 1J–L; Table 1).

**Table 1:**
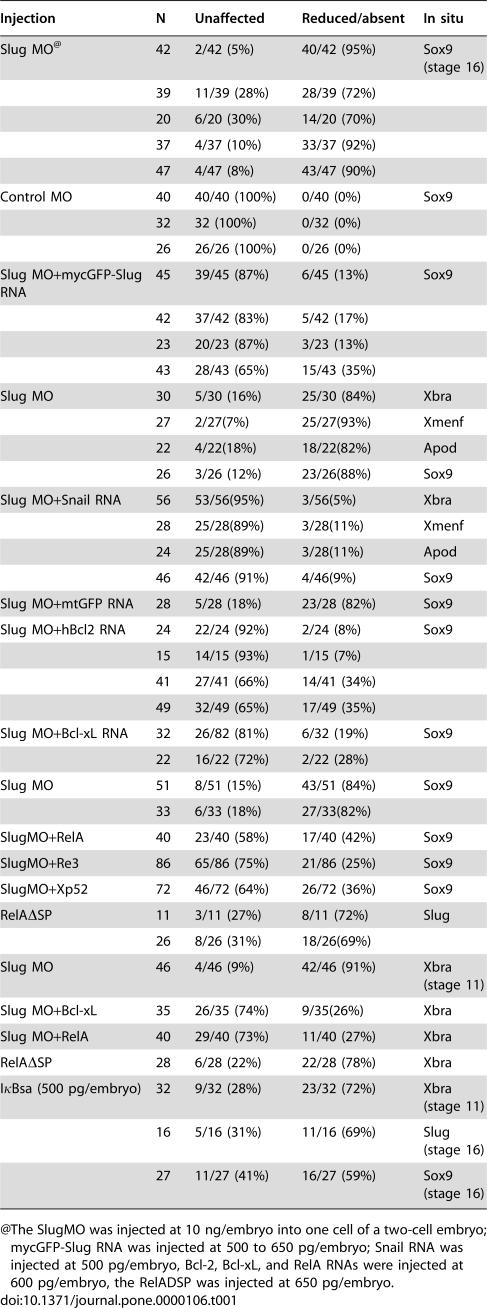
Slug Morpholino and Rescue experiments

Injection	N	Unaffected	Reduced/absent	In situ
Slug MO@	42	2/42 (5%)	40/42 (95%)	Sox9 (stage 16)
	39	11/39 (28%)	28/39 (72%)	
	20	6/20 (30%)	14/20 (70%)	
	37	4/37 (10%)	33/37 (92%)	
	47	4/47 (8%)	43/47 (90%)	
Control MO	40	40/40 (100%)	0/40 (0%)	Sox9
	32	32 (100%)	0/32 (0%)	
	26	26/26 (100%)	0/26 (0%)	
Slug MO+mycGFP-Slug RNA	45	39/45 (87%)	6/45 (13%)	Sox9
	42	37/42 (83%)	5/42 (17%)	
	23	20/23 (87%)	3/23 (13%)	
	43	28/43 (65%)	15/43 (35%)	
Slug MO	30	5/30 (16%)	25/30 (84%)	Xbra
	27	2/27(7%)	25/27(93%)	Xmenf
	22	4/22(18%)	18/22(82%)	Apod
	26	3/26 (12%)	23/26(88%)	Sox9
Slug MO+Snail RNA	56	53/56(95%)	3/56(5%)	Xbra
	28	25/28(89%)	3/28(11%)	Xmenf
	24	25/28(89%)	3/28(11%)	Apod
	46	42/46 (91%)	4/46(9%)	Sox9
Slug MO+mtGFP RNA	28	5/28 (18%)	23/28 (82%)	Sox9
Slug MO+hBcl2 RNA	24	22/24 (92%)	2/24 (8%)	Sox9
	15	14/15 (93%)	1/15 (7%)	
	41	27/41 (66%)	14/41 (34%)	
	49	32/49 (65%)	17/49 (35%)	
Slug MO+Bcl-xL RNA	32	26/82 (81%)	6/32 (19%)	Sox9
	22	16/22 (72%)	2/22 (28%)	
Slug MO	51	8/51 (15%)	43/51 (84%)	Sox9
	33	6/33 (18%)	27/33(82%)	
SlugMO+RelA	40	23/40 (58%)	17/40 (42%)	Sox9
SlugMO+Re3	86	65/86 (75%)	21/86 (25%)	Sox9
SlugMO+Xp52	72	46/72 (64%)	26/72 (36%)	Sox9
RelA*Δ*SP	11	3/11 (27%)	8/11 (72%)	Slug
	26	8/26 (31%)	18/26(69%)	
Slug MO	46	4/46 (9%)	42/46 (91%)	Xbra (stage 11)
Slug MO+Bcl-xL	35	26/35 (74%)	9/35(26%)	Xbra
Slug MO+RelA	40	29/40 (73%)	11/40 (27%)	Xbra
RelA*Δ*SP	28	6/28 (22%)	22/28 (78%)	Xbra
IκBsa (500 pg/embryo)	32	9/32 (28%)	23/32 (72%)	Xbra (stage 11)
	16	5/16 (31%)	11/16 (69%)	Slug (stage 16)
	27	11/27 (41%)	16/27 (59%)	Sox9 (stage 16)

@ The SlugMO was injected at 10 ng/embryo into one cell of a two-cell embryo; mycGFP-Slug RNA was injected at 500 to 650 pg/embryo; Snail RNA was injected at 500 pg/embryo, Bcl-2, Bcl-xL, and RelA RNAs were injected at 600 pg/embryo, the RelADSP was injected at 650 pg/embryo.

Maternal Slug RNA can be detected by RT-PCR [22 and data not shown] and is expressed zygotically in mesoderm [21]. The loss of Slug function during late bastula/early gastrula stages would be expected to influence both neural crest and placodal development, which depend upon signals from the mesoderm [66]–[70]. We generated plasmids that encode chimeric proteins consisting of glucocorticoid-binding regulatory domain [71], [72] and either Slug alone (GR-Slug) or Slug linked to a C-terminal GFP moiety (GR-Slug-GFP). In the absence of dexamethasone, both GR-Slug proteins are inactive and had no apparent effect on the Slug MO's ability to block *Sox9* expression (Table 2). When Slug MO and GR-Slug RNA injected embryos were exposed to dexamethasone beginning at stage 11, *Sox9* expression was efficiently recovered (Table 2).

**Table 2:**
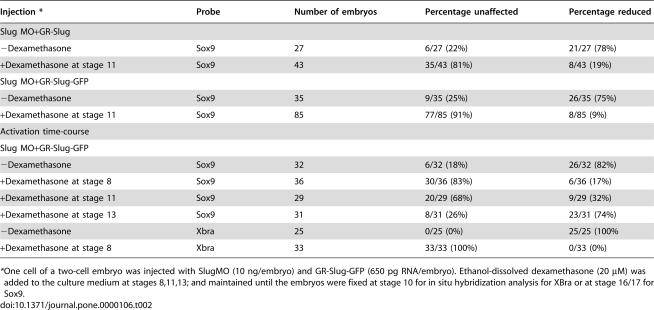
Timing of Slug Rescue

Injection *	Probe	Number of embryos	Percentage unaffected	Percentage reduced
Slug MO+GR-Slug				
−Dexamethasone	Sox9	27	6/27 (22%)	21/27 (78%)
+Dexamethasone at stage 11	Sox9	43	35/43 (81%)	8/43 (19%)
Slug MO+GR-Slug-GFP				
−Dexamethasone	Sox9	35	9/35 (25%)	26/35 (75%)
+Dexamethasone at stage 11	Sox9	85	77/85 (91%)	8/85 (9%)
Activation time-course				
Slug MO+GR-Slug-GFP				
−Dexamethasone	Sox9	32	6/32 (18%)	26/32 (82%)
+Dexamethasone at stage 8	Sox9	36	30/36 (83%)	6/36 (17%)
+Dexamethasone at stage 11	Sox9	29	20/29 (68%)	9/29 (32%)
+Dexamethasone at stage 13	Sox9	31	8/31 (26%)	23/31 (74%)
−Dexamethasone	Xbra	25	0/25 (0%)	25/25 (100%
+Dexamethasone at stage 8	Xbra	33	33/33 (100%)	0/33 (0%)

* One cell of a two-cell embryo was injected with SlugMO (10 ng/embryo) and GR-Slug-GFP (650 pg RNA/embryo). Ethanol-dissolved dexamethasone (20 µM) was added to the culture medium at stages 8,11,13; and maintained until the embryos were fixed at stage 10 for in situ hybridization analysis for XBra or at stage 16/17 for Sox9.

**Figure 2:**
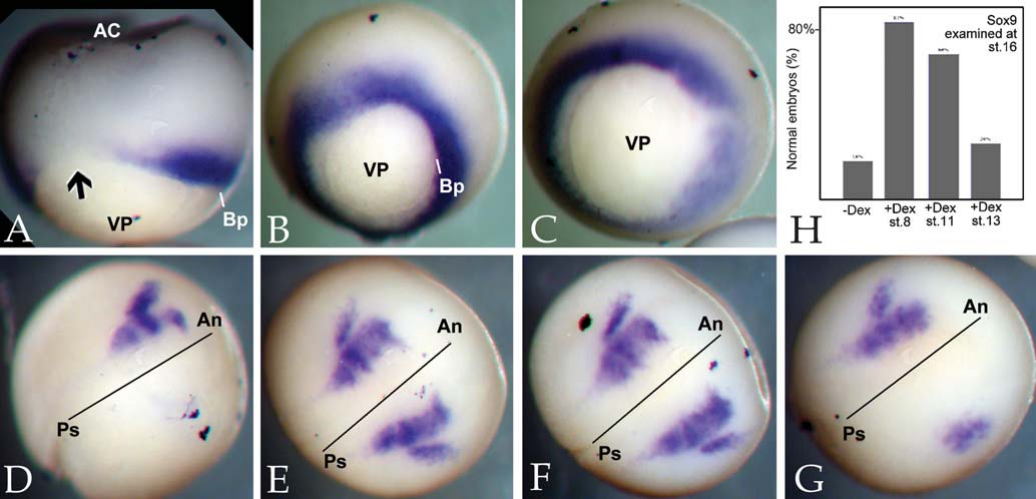
Timing of Slug rescue of Slug MO phenotypes: To analyze the timing of Slug activity in the early embryo, we injected one cell of two cell embryos with Slug MO (10 ng/embryo) together with RNA (650 pg/embryo) encoding the chimeric GR-Slug-GFP protein. A: In the absence of the activating drug dexamethasone, the Slug MO phenotype, i.e. suppression of *Xbra* expression in stage 11 embryos (A)(arrow) and suppression of *Sox9* expression in stage 16 embryos (D), was unaltered. When embryos were treated with dexamethasone (20 µM) beginning at stage 8, there as an essentially complete rescue of *Xbra* (B,C) and *Sox9* expression (E,H). Treatment of embryos with dexamethasone at stage 11 (early gastrulation) was also effective at rescuing *Sox9* expression (F,H), while addition of dexamethasone at stage 13 (late gastrulation/early neurulation) produced at most a partial and inefficient rescue of *Sox9* expression (G,H).

To examine the timing of Slug's role in neural crest formation, we compared the effects of activating the GR-Slug-GFP protein in mid-blastula (stage 8), early gastrula (stage 11), and late gastrula/early neurula (stage 13) embryos (FIG. 2; Table 2). Activation of Slug at stage 8 lead to a complete rescue of both mesodermal (*Xbra*) and neural crest/placodal (*Sox9*) marker expression. Efficient rescue of neural crest/placodal marker expression was also observed when Slug was activated at stage 11, but rescue was much less efficient when Slug was activated at stage 13 (FIG. 2; Table 2).

### Bcl-2/Bcl-xL suppression of the Slug MO phenotype

**Figure 3:**
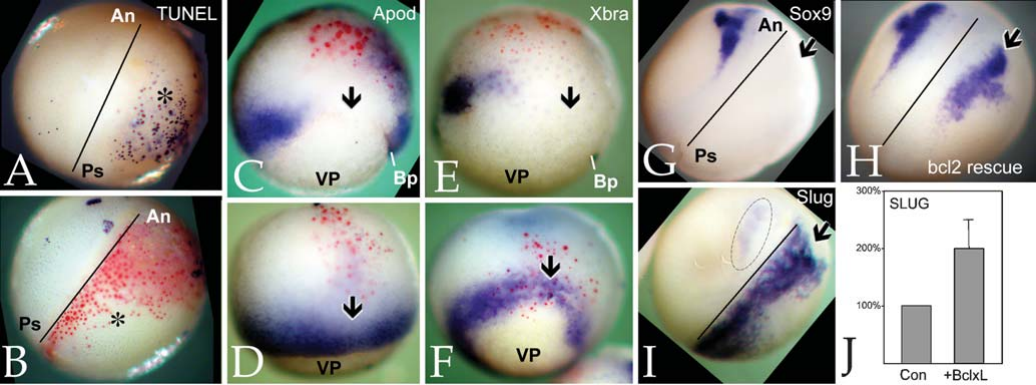
Rescue of Slug MO effects by Bcl-xL A: Injection of the Slug MO leads to an increase in TUNEL staining. B: This increase is blocked by the injection of Bcl-xL RNA (600 pg/embryo and co-injected with LacZ RNA). Injected sides of embryos are marked by an “*” and red staining; line marks midline of the embryo, with anterior (“An”) and posterior (“Ps”) indicated. Injection of Bcl-xL RNA (600 pg/embryo) rescues *Apod* (C- Slug MO injected, D-Slug MO+Bcl-xl RNA injected), *Xbra* (Ε- Slug MO injected, F-Slug MO+Bcl-xl RNA injected), and *Sox9* expression (G- Slug MO injected, H-Slug MO+Bcl-xL RNA injected). I: Injection of Bcl-xL RNA into one cell of a two-cell embryo led to a dramatic increase in the intensity and extent of *Slug* expression at stage 16; the region of Slug expression on the uninjected (control) side of the embryo is indicated by the dashed circle. J: Injection of Bcl-xL RNA produced an increase in Slug RNA levels in animal caps prepared at stage 8/9 and analyzed by QRT-PCR when uninjected embryos reached stage 11. Ornithine decarboxylase (ODC) was used as normalization control. RNA levels in the control case were set to 100%.

Inhibition of Slug activity by injection of Slug MO (FIG. 3A), antisense RNA (data not shown) or RNA encoding a dominant negative version of Slug (ZnfSlug) leads to increased numbers of apoptotic cells as visualized by TUNEL staining, while injection of Slug RNA suppresses apoptosis [61]. In our studies, carried out at stage 16/17, the increase in TUNEL positive cells was most prominent outside of the neural crest stage Slug expression domain, and so presumably represent effects on earlier developmental events.

To distinguish Slug's pro-EMT and anti-apoptotic activities, we injected embryos with RNAs encoding the anti-apoptotic proteins Bcl-2 (human) and Bcl-xL (*X. laevis*); both had similar effects and observations using Bcl-xL are show here. As expected, injection of Bcl-xL RNA blocked the Slug MO induced increase in TUNEL staining (FIG. 3B). Bcl-xL also rescued the expression of the early mesodermal markers *Apod* (FIG. 3C,D) and *Xbra* (FIG. 3E,F), the neural crest/otic placode marker *Sox9* (FIG. 3G,H; Table 1), and craniofacial morphology (data not shown). In stage 16/17 embryos, injection of Bcl-xL RNA led to a dramatic increase in the level and spatial extent of *Slug* expression (FIG. 3I). In ectodermal explants (animal caps), prepared at stage 8/9 from embryos injected with Bcl-xL RNA, and analyzed at stage 11 (∼3 hours later), there was a small (∼2×) but reproducible increase in the level of Slug mRNA, as determined by quantitative RT-PCR (FIG. 3J).

### Bcl-xL and Slug regulate NF-κB activity

**Figure 4:**
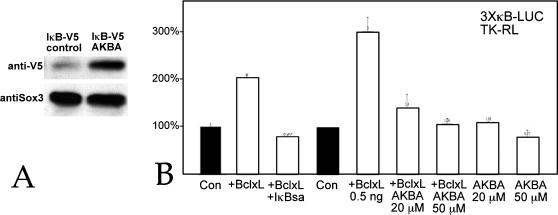
Characterization of Bcl-xL effects on NF-κB activity. A: Fertilized eggs were injected with RNA (650 pg/embryo) encoding Xenopus IκBα-V5. Beginning at stage 8, experimental embryos were treated with 50 µM AKBA and analyzed at stage 11 by SDS-PAGE/immunoblot using an anti-V5 antibody and the antiSOX3c antibody to visualize endogenous Sox3 protein as a loading control. AKBA treatment stabilized the IκBα-V5 polypeptide. B: Fertilized eggs were injected with p3XκB-firefly luciferase (“3κB-Luc”) and pTK-Renilla luciferase (“RL-TK”) DNAs (10 pg/embryo each) either alone (“Con”) or together with Bcl-xL (500 pg/embryo) RNA, or Bcl-xL and IκBsa (600 pg/embryo) RNAs. Alternatively, animal caps prepared from Bcl-xL RNA injected embryos were cultured in either control buffer (0.1% DMSO), 20 µM or 50 µM AKBA. At stage 11, caps were analyzed for luciferase activity. Bcl-xL induced an increase in 3XκB-Luc activity that was blocked by either IκBsa or AKBA. Error bars in B reflect standard deviation from the mean of multiple experiments.

Bcl-2 and Bcl-xL are structurally similar cytoplasmic proteins. Bcl-2 can influence gene expression through effects on IκB kinase activity (IκK)[73]–[77]. To examine Bcl-xL's effects on NF-κB activity in Xenopus, we used the NF-κB responsive p3XκB-Luc reporter plasmid together a mutated form of human IκBα (IκBsa) and acetyl-11-keto-β-boswellic acid (AKBA). In IκBsa serines 32 and 36, normally phosphorylated by IκK, are mutated to alanines. The stable IκBsa polypeptide acts as a dominant repressor of NF-κB activity [78]. AKBA inhibits IκK activation [79]–[81]. Treating animal caps with 50 µM AKBA stabilized an epitope-tagged form of *Xenopus* IκBα (FIG. 4A). Fertilized eggs were injected with p3XκB-Luc plasmid DNA, pTK-Renilla luciferase plasmid DNA (as a normalization control), and Bcl-xL RNA alone or together with IκBsa RNA; animal caps were prepared and analyzed for luciferase activity at stage 11. Alternatively, fertilized eggs were injected with p3XκB-Luc and pTK-Renilla DNAs and Bcl-xL RNA, animal caps were prepared and then incubated in control media or in media containing AKBA. Bcl-xL increased NF-κB reporter activity and this increase was blocked by both IκBsa and AKBA (FIG. 4B). On its own, AKBA had little effect on reporter activity.

### Bcl-xL and Slug regulation of Rel expression

**Figure 5:**
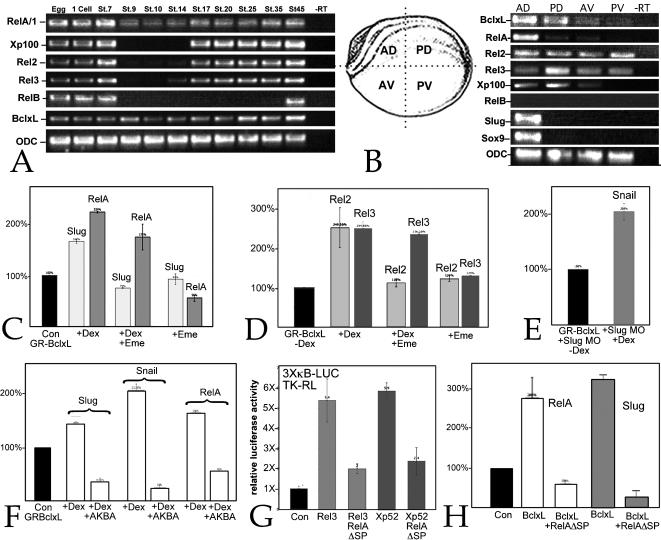
Bcl-xL regulation of NF-κB RNAs: A: RNA was extracted from eggs and embryos at various stages and analyzed by RT-PCR (28 cycles); levels of RelA, Rel2, Rel3, RelB and Xp100 RNAs drop between stage 7 and 9 and, except for RelB, increase following gastrulation (stage 12/13). Levels of Bcl-xL RNA appear relatively constant throughout this period of development. B: At stage 16, embryos were dissected into anterior dorsal (AD), posterior dorsal (PD), anterior ventral (AV), and posterior ventral (PV) quadrants and RNA was analyzed by RT-PCR; *RelB* was not expressed at this stage; expression of *RelA*, *Slug* and *Sox9* are restricted to the anterior dorsal quadrant, while Bcl-xL, Rel2, Rel3, and Xp100 RNAs can be detected throughout the embryo. C, D: Animal caps were prepared from embryos injected with GR-Bcl-xL-GFP RNA (“GRBclxL”)(600 pg/embryo) and either left untreated (0.1%DMSO)(“Con”), treated with 20 µM dexamethasone (“+Dex”), treated first with 100 µg/ml emetine and then dexamethasone (“+Dex+Eme”), or treated with emetine alone (“+Eme”), and analyzed at stage 11 for Slug, RelA (C), Rel2 and Rel3 (D) RNA levels. Treatment with emetine blocked the increase in Slug and Rel2, but not RelA and Rel3 RNAs; emetine treatment alone produced control or slightly reduced levels of Slug and Rel RNAs. E: Activation of the GR-Bcl-xL-GFP protein in embryos injected with the Slug MO produces an increase in the level of Snail RNA, analyzed at stage 11. F: In animal caps derived from GR-BclxL-GFP RNA injected embryos, AKBA (50 µM) inhibited the dexamethasone-induced increases in Slug, Snail, and RelA RNA levels; while treatment with AKBA alone lead to a decrease in Slug, Snail and RelA RNA levels. G: In animal caps, injection of RelAΔSP RNA (600 pg/embryo) blocked Rel3 and Xp52 RNA induced activation of the 3XκB reporter. H: The ability of Bcl-xL RNA to increase levels of RelA and Slug RNAs in animal caps was blocked by the co-injection of RelAΔSP RNA. Error bars in C–H reflect standard deviation from the mean of multiple experiments.

Five NF-κB subunit genes have been characterized in *X. laevis*: RelA/Rel1 [82], [83], Rel2 [84], Rel3 [85], RelB [86] and Xp100 [87]. All five RNAs are supplied maternally and their levels drop at the onset of zygotic transcription (stage 8/9)(FIG. 5A). As development proceeds Rel2, Rel3 and Xp100 RNAs are again detectable by RT-PCR (28 cycles), while RelB RNA does not reappear until after stage 35 [86]. Bcl-xL RNA levels appear constant throughout this period [88](FIG. 5A). In stage 16/17 embryos, RelA, Rel2, Rel3, and Xp100 RNAs can be readily detected by RT-PCR in the anterior-dorsal quadrant of the embryo; the same region where *Slug* and *Sox9* are normally expressed (FIG. 5B). RelA appears concentrated in the anterior dorsal sector, while Rel2 and Rel3, and to a lesser extend Xp100 appear to be present at similar levels throughout the embryo.

To explore the mechanism of Bcl-xL regulation of Slug and NF-κB, we generated a plasmid encoding a glucocorticoid-binding domain-Bcl-xL-GFP (GR-BclxL-GFP) chimera. In the absence of dexamethasone, GR-BclxL-GFP does not alter Slug, RelA, Rel2 or Rel3 RNA levels, while addition of dexamethasone leads to their increase (FIG. 5C,D), similar to that seen using the non-hormone regulated form of Bcl-xL (FIG. 3J). No effect was observed on Xp100 RNA levels (data not shown). While these effects are small, i.e., 2–3 fold, they are highly reproducible. Adding the protein synthesis inhibitor emetine blocks the dexamethasone-dependent increase in Slug and Rel2 RNA levels, but not the increase in RelA or Rel3 RNA levels; emetine alone had no reproducible effect on RNA levels (FIG. 5C,D). These results suggest that Bcl-xL acts directly to regulate RelA and Rel3, and indirectly to regulate Slug and Rel2 RNA levels. By “direct” we mean a regulatory interaction that does not require on-going protein synthesis (see Discussion). Bcl-xL activation also leads to an increase in the level of Snail RNA (FIG. 5E); this increase does not appear to involve effects on Slug RNA, since it occurs in the presence of the Slug morpholino.

AKBA treatment blocked Bcl-xL's ability to increase levels of Slug, Snail and RelA RNAs (FIG. 5F), suggesting that Bcl-xL acts on these RNAs, as it does on the NF-κB responsive reporter, by increasing IκB kinase activity. RelA*Δ*SP is a dominant negative form of RelA [89]; it dimerizes with other NF-κB subunit proteins and blocks their activity. When co-injected with RNAs encoding Rel3 or Xp52, the active form of the NF-κB subunit protein Xp100 [87], RelAΔSP inhibited their ability to activate of the 3XκB-Luc reporter (FIG. 5G) and inhibited Bcl-xL's ability to increase RelA and Slug RNA levels (FIG. 5H), indicating that active NF-κB is required for Bcl-xL to induce increases in Slug and RelA RNA levels.

**Figure 6:**
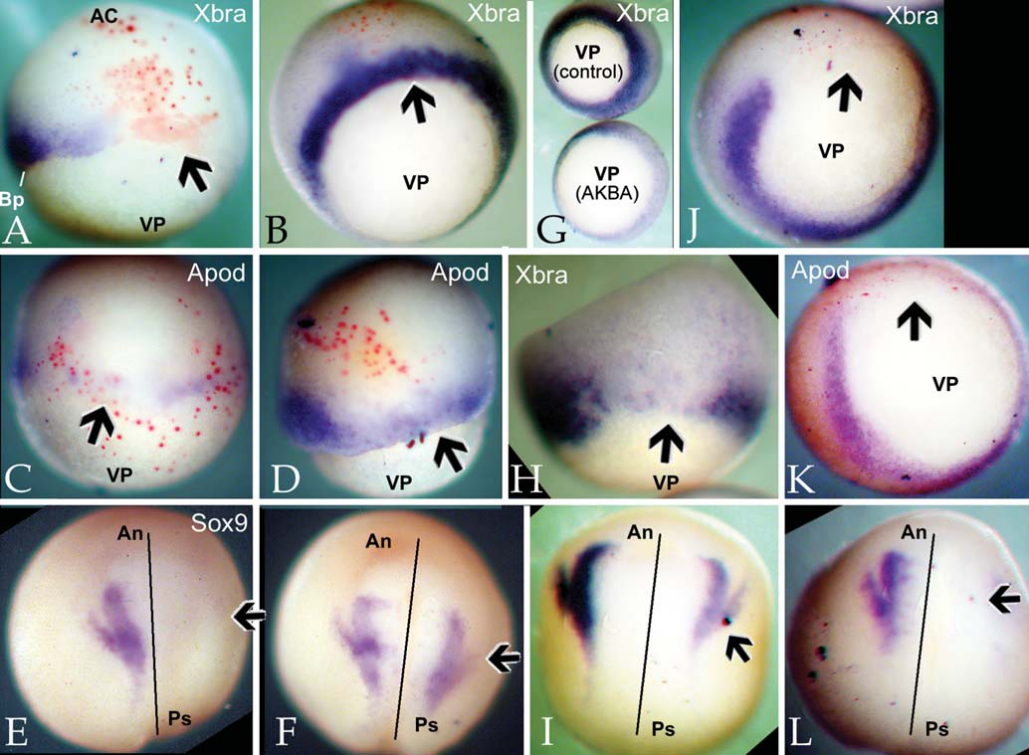
NF-κB regulation of mesodermal and neural markers: The Slug MO induced loss of *Xbra* (A,B), *Apod* (C,D) and *Sox9* (E,F) expression was rescued by injection of RelA RNA (600 pg/embryo)(A, C, E-Slug MO alone, B, D, F-Slug MO+RelA RNA). G: Treatment of early embryos with AKBA (50 µM from the 4-cell stage on) lead to a decrease in *Xbra* staining (control and AKBA-treated embryos marked). Injection of RNA encoding IκBsa (H,I) or RelAΔSP (J–L) had effects similar to that seen in Slug MO injected embryos; that is, both induced the reduction of Xbra (H,J), Apod (K) and Sox9 (I,L) RNA staining. AKBA treatment had no reproducible effect on *Sox9* expression (data not shown). Arrows mark affected regions.

Assuming that Bcl-xL acts to rescue the effects of the Slug MO through its ability to regulate NF-κB activity, injection of RelA RNA should be able to rescue the Slug MO phenotype. In embryos injected unilaterally with the Slug MO, injection of RelA RNA lead to re-appearance of both Xbra (FIG. 6A,B) and Apod (FIG. 6C,D) RNAs. In later stage, Slug MO-injected embryos, injection of RelA RNA lead to the reappearance of Sox9 expression in both the neural crest and otic placode regions, (FIG. 6E,F). A similar rescue of Sox9 expression in Slug MO injected embryos was observed upon injection of Rel3, or Xp52 RNAs (600 pg/embryo)(Table 1). We have not examined RelA's effects on Slug RNA in Slug MO injected embryos because morpholinos typically stabilize, rather than induce the degradation of, their target RNAs (unpubl. obs.); RelA does induce an increase in Slug RNA levels, as monitored by *in situ* hybridization, when injected on its own (data not shown – see below).

To complement these studies, we examined the effects of treating embryos with AKBA or injecting one cell of two cell embryos with either IκBsa or RelAΔSP RNAs. AKBA treatment, beginning at the 4-cell stage, led to a noticeable decrease in the intensity of Xbra RNA staining in early gastrula stage embryos (FIG. 6G), but had little reproducible effect on Sox9 RNA levels in neurula stage embryos (data not shown). Injection of IκBsa RNA lead to the suppression of *Xbra* (FIG. 6H; Table 1) and the reduction of *Sox9* (FIG. 6I) expression. Injection of RelAΔSP RNA inhibited expression of *Xbra* (FIG. 6J), *Apod* (FIG. 6K), and *Sox9* (FIG. 6L).

### NF-κB regulation of Slug

**Figure 7:**
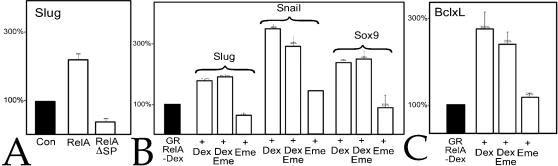
NF-κB's regulatory targets: A: In animal caps, RelA lead to an increase in Slug RNA levels, while RelA*Δ*SP produced a decrease. When activated by dexamethasone (+Dex), the hormone-regulated form of RelA, GR-RelA (600 pg RNA/embryo), induced a similar increase in the levels of Slug RNA, as well as Snail, Sox9 (B), and Bcl-xL (C) RNAs compared to animal caps from GR-RelA injected embryos not exposed to dexamethasone. Similar effects were seen in the presence of emetine (+Dex+Eme,), while emetine alone (+Eme) had little effect on any of measured RNA levels. Error bars in reflect standard deviation from the mean of multiple experiments.

To examine whether of NF-κB directly regulates Slug and Snail RNA levels, we first characterized the effects of RelA and RelA*Δ*SP in animal caps; injection of RelA RNA lead to an increase, while RelA*Δ*SP RNA lead to a decrease in Slug RNA levels (FIG. 7A). Using a dexamethasone-regulated form of RelA, GR-RelA, we found a similar effect – in the presence of dexamethasone GR-RelA produced an emetine-insensitive increase in Bcl-xL (FIG. 7B), Slug, and Snail RNAs (FIG. 7C). Given RelA's ability to induce *Sox9* expression in Slug MO injected embryos (see above), we examined RelA's effect on Sox9 RNA levels; activation of RelA led to an increase Sox9 RNA levels even in the presence of emetine (FIG. 7C).

### Targets of Slug regulation

**Figure 8:**
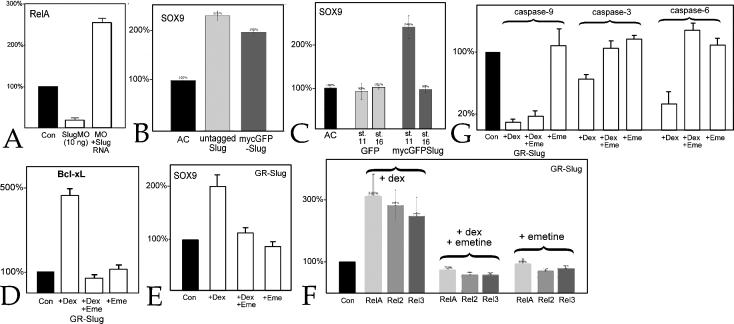
Slug's regulatory targets: A: In animal caps analyzed at stage 11, the Slug MO (10 ng/embryo) produced a decrease in RelA RNA levels that was rescued by co-injection of mycGFP-Slug RNA (1 ng/embryo). B: Animal caps were prepared from embryos injected with either untagged or mycGFP-Slug RNAs; both produced a similar increase in Sox9 RNA levels. C: Animal caps, from embryos injected with either mt-GFP or mycGFP-Slug RNAs, were analyzed when control embryos reached stage 11 or stage 16; at stage 11 mycGFP-Slug induced an increase in Sox9 RNA levels, which returned to baseline by stage 16. No change in Sox9 RNA levels were observed at either stage in animal caps expressing mt-GFP. In GR-Slug injected caps, levels of Bcl-xL (D), Sox9 (E), RelA, Rel2 and Rel3 (F), and caspase-9, -3 and -6 (G) RNAs were increased in response to dexamethasone; with the sole exception of caspase-9, these increases were blocked by emetine. In all panels, error bars reflect standard deviation from the mean of multiple experiments.

In *Drosophila* *Dorsal*, which encodes a RelA homolog, regulates *Snail* expression, and Snail in turn regulates *Dorsal* expression [see 90], [91]. In animal caps, the Slug MO decreased and mycGFP-Slug increased RelA RNA levels (FIG. 8A), suggesting an analogous regulatory circuit. To characterize Slug's interactions with regulatory targets, we used the GR-Slug construct; its activity in the presence of dexamethasone is similar to that of mycGFP-tagged and untagged versions of Slug. In animal caps, both mycGFP-Slug and dexamethasone-activated GR-Slug lead to increased levels of the neural crest marker *Zic5* [92], [93](data not shown) and *Sox9* (FIG. 8B). In contrast Aybar et al., [23] reported that Slug did not induce neural crest markers in animal caps analyzed at stage 20, ∼22 hours after fertilization. To reconcile these observations, we analyzed animal caps derived from embryos injected with Slug RNA at stage 11 (our standard analysis time point) and stage 16; *Sox9* RNA levels were increased at stage 11 but had returned to control levels by stage 16 (FIG. 8C), indicating that factors in addition to Slug are required to maintain *Sox9* expression. In independent studies we have found that levels of Sox3 and SoxD RNAs, whose expression is associated with early germ layer and neural differentiation, change dramatically in the period between stage 11 and 14 (C. Zhang, T. Grammer & M.W. Klymkowsky, unpubl. obs). GR-Slug positively but indirectly increased levels of Bcl-xL (FIG. 8D), Sox9 (FIG. 8E), RelA, Rel2, and Rel3 RNAs (FIG. 8F), but had no effect on Xp100 RNA levels (data not shown).

*Caspase-9* encodes an initiator caspase involved in the maternal/early embryonic apoptotic program in *X. laevis*, while the effector caspases-3 and -6 act downstream [94]. *Caspase-9* appears to be a direct and negatively regulated target of Slug, while *caspases-3* and *-6* appear to be indirect targets (FIG. 8G). In embryos, Slug MO induced an increase in caspase activity as indicated by staining with the anti-activated caspase antibody CM1 and increased cleavage of a caspase-3 target peptide (data not shown). These results extend those of Tribulo et al., [61] and establish, apparently for the first time, a direct regulatory interaction between Slug and caspase-9.

## Discussion

**Figure 9:**
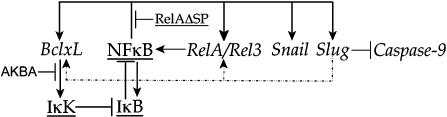
Bcl-xL-Slug-NF-κB network: This diagram focuses on the regulatory interactions uncovered in the course of our studies (see text for caveats associated with the identification of direct interactions). Protein names are underlined, gene names are in italics. Bcl-xL appears to activate NF-κB through effects on IκK activity and IκB stability. NF-κB acts directly to regulate Slug, Snail, RelA, and Rel3 levels; NF-κB regulation of the expression of its inhibitor IκB is based on data from mammalian systems. *Caspase-9* was the only direct target of Slug identified in our studies; indirect interactions are indicated by dotted lines.

In analogy with polymerization reactions, scientific studies often involve distinct initiation and catalytic events. In this work, the initiator was the observation that anti-apoptotic proteins rescue the effects of blocking *Slug* expression on mesodermal and neural crest markers. Bcl-xL produced an increase in both *Slug* and *Snail* RNAs and Snail itself is sufficient to suppress the Slug morpholino phenotype (FIG. 1J–L)[22]. The role of catalyst was played by the observations of Kirshenbaum and colleagues [XPATH ERROR: unknown variable "start".]–, who found that Bcl-2 regulates NF-κB activity by activating IκB kinase (IκK). Activation of IκK induces the degradation of inhibitory IκB proteins, leading to increased NF-κB activity. Our studies using the dominant negative IκBsa protein, the IκK inhibitor AKBA, and the dominant negative form of RelA, RelAΔSP, indicate that Bcl-xL regulates Slug RNA expression via NF-κB in the early *X. laevis* embryo (FIG. 9).

### Mapping regulatory interactions

In mammalian epithelial cells over-expression of Bcl-2 has been found to promote EMT [96] and suppress cadherin expression [97]. Neither study, however, examined the effects of Bcl-2 expression on the levels of Slug or Snail RNAs. NF-κB itself has been implicated in EMT [98], and has been found to regulate Snail stability and activity through effects on glycogen synthase kinase 3 [99]. In *X. laevis*, few promoters have been rigorously defined. It is possible, however, to tentatively classify regulatory interactions as direct or indirect based on the ability of hormone-regulated proteins to influence target RNAs in the presence of protein synthesis inhibitors. If a regulatory interaction requires or depends upon on-going protein synthesis, it is classified as “indirect”; “direct” interaction are not blocked by protein synthesis inhibitors.

Regulatory interactions are often complex and multifaceted; it is important to remember that conclusions based on hormone-regulated proteins need to be characterized further. For example, if a transcription factor regulates the expression of a gene encoding a microRNA, which in turn regulates the stability of a target RNA, its effects will appear as direct even though they are mechanistically indirect. As another example, NF-κB acts as both a transcriptional regulator [100] and has been reported to destabilize certain RNAs [101]. This latter activity would appear as direct in our system. In this light it is interesting to note that activation of GR-RelA leads to a protein-synthesis independent decrease in levels of p53 RNA (unpubl. obs); whether this reflects transcriptional, post-transcriptional, or microRNA-mediated regulation is not yet resolved.

Neither Bcl-2 or Bcl-xL are thought to regulate gene expression through direct interactions with DNA or transcription factors, but rather through effects on various kinases [see 74]–[76]. Using a hormone-regulated form of Bcl-xL, *RelA* and *Rel3* appear to be direct, while *Rel2* and *Slug* appear to be indirect targets of Bcl-xL regulation. The ability of Bcl-xL to induce changes in RelA, Slug and Snail RNA levels is inhibited by both the dominant negative form of RelA, RelAΔSP and the IκK inhibitor AKBA, suggesting that Bcl-xL activates IκK, which initiates the destruction of IκB polypeptides, leading to the activation of pre-existing NF-κB, which in turn regulates target genes (FIG. 9). The presence of RelAΔSP or AKBA blocks these NF-κB-dependent processes and so blocks the effects of Bcl-xL on both “direct” and “indirect” targets.

### NF-κB regulation of Slug

NF-κB subunit proteins are expressed ubiquitously and play a key role in cellular inflammation and tumor progression [102]–[106]. NF-κB is known to have a number of regulatory targets, including IκBα [107], [108], which acts to repress, and so limit, NF-κB activity. In mammalian systems, NF-κB regulates the expression of a range of anti-apoptotic proteins, including the anti-apoptotic caspase inhibitor proteins (IAPs), Bcl-2, and Bcl-xL [109]–[113] and decreases the activity of the pro-apoptotic p53 protein in renal cell carcinoma cells [114]. In addition, NF-κB and p53 can inhibit each other's activities by competing for the limited pool of CBP/p300 within the cell [115]. In *X. laevis* RelA/NF-κB is a positive regulator of *Bcl-xL*, *Slug* and *Snail*. Slug's ability to down-regulate the pro-apoptotic genes *Caspase-9* [61] and *Puma* [45] would be expected to generate an over-all anti-apoptotic state.

In this light, the decrease in NF-κB RNA levels at the midblastula transition (FIG. 5A) may be permissive in the regulation apoptotic processes in later stage embryos [116]–[118]. A number of drugs, e.g. AKBA and curcumin [79], [81], [119]–[121], inhibit NF-κB activity and increase apoptosis, perhaps by reducing levels of Slug and/or Snail expression, which may explain at least part of their anti-tumor effects.

### Slug regulation of NF-κB

In *X. laevis* Slug activates an NF-κB responsive reporter and acts indirectly to increase levels of RelA, Rel2, and Rel3 RNAs. In *Drosophila* Snail also acts indirectly to regulate *Dorsal* (RelA) by inhibiting expression of *WntD,* which acts to inhibit activation of *Dorsal* [90], [91]. How Slug regulates RelA/Rel3 expression in Xenopus remains unclear, but preliminary studies indicate that *Drosophila* WntD, as well as a number of Xenopus Wnts, inhibit Bcl-xL-mediated activation of the 3XκB-Luc reporter and reduce RelA RNA levels (Zhang & Klymkowsky, unpubl. obs.). Whether Slug acts through the regulation of a Wnt or some other intermediate, it is apparent that Slug can increase NF-κB activity and RNA levels in the early Xenopus embryo. Our studies indicate that NF-κB regulates both Slug and Snail RNA levels and plays an essential role in mesoderm formation. The presence of a heretofore unrecognized NF-κB–Slug/Snail regulatory loop in a vertebrates should have important consequences for our understanding the conserved and divergent evolutionary mechanisms involved in germ layer specification, as well as practical implications for therapeutic interventions that target NF-κB and Slug/Snail-mediated EMT and anti-apoptotic processes.

## Materials and Methods

### Embryos and animal caps

*X. laevis* embryos were obtained following standard protocols [22], [72] from adult animals purchased from Xenopus I, Inc. (Dexter, MI). Embryos were staged according to Nieuwkoop and Faber [122]. Fertilized eggs or one-cell of two-cell embryos were injected with 10–20 nL of solution; ectodermal explants (animal caps) were prepared from stage 8/9 embryos using a Gastromaster™ (Xenotech) and cultured until control embryos reached either stage 11 or stage 16/17 [72], [123]. In experiments involving hormone activation of chimeric polypeptides, whole embryos or animal caps were treated with 20 µM dexamethasone (Sigma) alone, or were pretreated for 30 minutes with 100 µg/mL of the protein synthesis inhibitor emetine (Sigma)[124], prior to dexamethasone and emetine treatment. In contrast to cycloheximide [125], emetine does not induce nodal gene expression under these conditions [126], [127]. RNA was isolated and subjected to either standard or quantitative RT-PCR (QRT-PCR) analysis as described previously [123]. Primers for PCR analyses were:

*caspase-3* [F5′AAGTCTGGAACATCGCAGG3′; R5′TAAATGAGCCCCTCATCACC3′];

*caspase-6* [F5′TGGACATCAAGGACTGTGGA3′; R5′CTGAACATCAAACCCCAGGT 3′];

*caspase-9* [F5′CCGATGGAGTTTCAAGCAAA3′; R5′GACTGGGCAGAAGGATTCAG3′];

*Bcl-xL/Xr11* [F5′GTCGGCCTGTATGGAAAGAA3′; R5′CATGATAGGCGACCCAGTG3′] [61];

*Slug* [F5′CAATGCAAGAACTGTTCC3′; R5′TCTAGGCAAGAATTGCTC3′];

*Snail* [F5′AAGCACAATGGACTCCTT3′; R5′CCAATAGTGATACACACC3′][22];

*Sox9* [F5′GAGAATGGTAGGCAGCCACCTCGC3′;

R5′CTGTTGCTGTTGGTCACTGTAATG3′](this study);

*RelA* [F5′GCGGATCCGAAGGGCGCTCTGCTGGAAGC3′;

R5′GCGAATTCAATTTCATCTCCTCCCAAGCA3′][86];

*Rel2* [F5′GCAGTTCCATCACAGCTAAAC3′; R5′GGTGTCTGGTAGCCTTTGGTC3′];

*Rel3* [F5′ATCATGGAAAGTTTGAGGGCA3′; R5′GGGTGGTAACTAAATGGTGTA3′];

*RelB* [F5′CCTCAGTACTGTAACTGTCGC3′; R5′GCAGTCTTTACCTACAAGGCC3′];

*Xp100* [F5′CTGATAAACATGCCAGATTAC3′; R5′GCACATCAGAGTCACTCTCAG3′](this study.

### Plasmids, morpholinos, and reporters

pCS2mycGFP-Slug and pCS2mt-GFP have been described previously [22]. Plasmids encoding dexamethasone-regulated versions of Slug, RelA and Bcl-xL were generated by subcloning into the pCS2GR-Sox7-GFP plasmid [127]. A plasmid encoding an epitope-tagged form of Xenopus Bcl-xL (Xr11)[88] was supplied by James Maller (UCHSC, Denver, CO); the pCS2-LacZ plasmid was supplied by Jing Yang (Columbus Children's Research Institute); a plasmid encoding human Bcl-2 was supplied by Jean Gautier (Columbia University); plasmids encoding Xenopus RelA(Rel1), myc-tagged Rel3, Xp52 (the active form of Xp100), and a dominant-negative form of RelA, RelAΔSP, were supplied by Hugh Woodland (U. Warwick)[83]Beck et al 1998), Ken Kao (U. Newfoundland)[85], [128], [129], and Jun-ichiro Inoue (U. Tokyo)[86], [87]. The Xp52 sequence was subcloned into pCS2 to form pCS2-Xp52. The p3XκB-Luc plasmid, which contains three κB binding sites driving expression of firefly luciferase [130] and a plasmid encoding a form of human IκBα (IκBsa) in which serines 32 and 36 have been mutated to alanines [78] were supplied by Lorrie Kirshenbaum (U. Manitoba). IκBsa is resistant to IκB kinase phosphorylation and subsequent proteolytic degradation, and so acts as a dominant-negative regulator of NF-κB activity [75], [78]. The coding sequence for *X. laevis* IκBα (GENBANK Accession AAH77876) was isolated by RT-PCR and subcloned into a pCS2-V5 plasmid to form pCS2-XIκBα-V5. Acetyl-11-keto-β-boswellic acid (AKBA), a pentacyclic triterpene, inhibits IκBα phosphorylation and degradation in mammalian systems [79]–[81]; it has also been reported to inhibit topoisomerases [131], [132] and 5-lipoxygenase [133]. The effects of AKBA on IκBα-V5 stability in *Xenopus* were analyzed using SDS-PAGE/immunoblot with an monoclonal anti-V5 epitope antibody (Invitrogen) and the antiSOX3c antibody [123]. Reporter assays were carried out using the dual luciferase system [123]. Capped RNAs were generated using Ambion mMessage mMachine kits. Both fluorescein-conjugated and unconjugated forms of a morpholino directed against the 5′ UTR and coding sequence of the *SlugA* and *SlugB* genes (FIG. 1A) [5′CGTGGCATTTTCACTGCGGGCGGGA3′] were used with identical results; these and a control morpholino were purchased from Gene Tools, Inc.

### TUNEL, anti-caspase staining and caspase cleavage assays

Fixed and sectioned embryos [134] were stained by TdT-mediated dUTP-biotin nick end-labeling (TUNEL) using a peroxidase-based kit purchased from Molecular Probes, following the manufacturer's instructions. Whole-mount TUNEL [135] was carried out using the protocol on the Harland Lab website^1^. The rabbit anti-activated caspase 3 antibody CM1 (BD Bioscience Pharmingen) was used in whole-mount immunocytochemistry at a dilution of 1∶1000 following standard immunocytochemical techniques [136], [137]. For caspase cleavage assays, embryo lysates were prepared and reactions were carried out in duplicate using one embryo equivalent of lysate (20 µL) and 80 µL lysis buffer. Reactions were incubated at 37°C for 1 hour with 5 µM of the caspase-3 fluorogenic substrate Ac-DEVD-AMC (BioMol), after which 990 µL of water was added and fluorescence was measured using a Hitachi F2000 Fluorescence Spectrophotometer. Results were analyzed for statistical significance using Student's t-test of the means.

### *In situ* hybridization and Alcian Blue Staining

Plasmids containing the *Sox9* coding sequence, isolated by RT-PCR from neural stage embryos (Fawcett & Klymkowsky, unpublished). Digoxigenin-labeled antisense probes were generated against Sox9, Slug [70], Sox2 [138], Sox3 [139], epidermal keratin [140], Xbra [141], Antipodean (Apod) [142], and Xmenf [143] RNAs and *in situ* hybridization was performed following standard protocols [22], [72]. Alcian Blue staining was carried out as described previously [134]. Digital images were captured using a Nikon CoolPix 995 Camera on an Inverted Leica M400 Photomicroskop. Images were manipulated with Fireworks 8 software (Macromedia now Adobe) using the “auto levels” and “curves” functions only.

## Footnotes

1
http://tropicalis.berkeley.edu/home/gene_expression/TUNEL/TUNEL.html
